# Supplementary material for: Investigating the coupling relationships of railway safety risks using the N-K model and complex network theory
Source: PLoS One. 2025 Sep 22;20(9):e0332948. doi: 10.1371/journal.pone.0332948 (PMC12453224; doi:10.1371/journal.pone.0332948)
Supplement: Attachment — Submitted filename: Response to Reviewers.docx [file pone.0332948.s002.docx]

Dear editor and reviewers,

We sincerely thank the editors and all the reviewers for their valuable comments, which have helped us improve the quality of the manuscript. The reviewers' comments are presented in *italic font*, with specific issues numbered. Our responses are presented in normal font, while the modifications and additions to the manuscript are marked in **blue font**. Additionally, in the revised manuscript, the modified charts, formulas, and symbols are highlighted in **yellow**.

**Editor:**

Thank you for your comprehensive guidance and the numerous requirements you have specified. We have meticulously reviewed and incorporated all essential modifications. The detailed response is presented as follows:

**1)Regarding the PLOS ONE style requirements and file naming**

Following the style template you provided, we have thoroughly revised the manuscript, including the main text format, title, and author information. Adjustments have been made to the font, paragraph layout, and chart annotations to ensure alignment with the journal's specifications.

**2) Regarding code sharing**

All analytical results in this study were derived using the transparent formulas presented in the manuscript (such as the coupling value calculation for the N-K model and node centrality computation) along with conventional statistical methods. No custom code or programming implementation was utilized. All quantitative results can be replicated manually by substituting the data into the provided formulas, allowing readers to independently verify the conclusions without requiring access to code. Consequently, there is no author - generated code to share in this study. The reproducibility of the analytical process has been ensured through methodological transparency, which aligns with the journal's requirements for research reproducibility.

**3) Regarding the revised funding statement**

We have revised the funding statement to comprehensively encompass all financial sources. The updated statement reads as follows: "This research, as well as the writing and publication of the article, was funded by the Science and Technology Research and Development Program of China Railway Corporation (Approval Number: K2024T002). No other external funding was received for this study." This statement fully reflects all relevant support. Thank you for your assistance in updating the online submission form.

**4) Declaration of Data Availability**

The data utilized in this study are the property of the Railway Safety Research Center, China State Railway Group Co., Ltd. Researchers interested in obtaining accident and fault data should contact the Railway Safety Research Center directly. The relevant contact information is: (86) 010-51871181. The authors do not possess any special access rights. All other data necessary for the study are fully included within the manuscript.

**5) Regarding the deletion of financial information in the acknowledgments section and the update of the funding statement**

We have removed the financial - related content from the acknowledgments section to ensure that the funding information is exclusively presented in the funding statement. The updated funding statement is consistent with the revision detailed in point 3. We kindly request your assistance in updating the online form accordingly.

**Reviewer 1:**

Thank you for your decision and constructive comments on my manuscript. We agree with the reviewers' suggestions and will incorporate the recommended changes into the manuscript.

***Major Issues/Suggestions for Improvement:***

**1) *The paper specifically needs improvement in the methodology section where many things are not clearly mentioned. For instance,***

**i) *how was the literature research and data collection done to search for accident reports? Was any database searched or was any other approach adopted?***

Your attention to the details of the literature research and data collection methods is greatly appreciated. The literature review and accident report collection for this study were conducted based on existing public resources and standard procedures. The following is the specific implementation path to demonstrate the systematic nature of the process and the reliability of the data:

1)Specific Implementation of Literature Research

The literature research concentrated on two primary directions: "Railway Risk Coupling" and "Application of Network Analysis". It was based entirely on existing academic databases:

Database Selection: Well - recognized Chinese and English databases in the field of safety science were employed to ensure comprehensive coverage of core domestic and international research.

Search Strategy: A combined keyword search was used, including terms such as "Railway Accident Risk", "Risk Factor Coupling", "N - K Model Safety Analysis", and "Complex Network Risk Transmission".

2)Collection and Screening of Accident Report Data

The focus was on cases of "Multi - factor Synergistic Hazard", such as those involving both human operation and equipment defects. A total of 157 sample cases were obtained, covering four factors: personnel, equipment, environment, and management. These cases closely aligned with the research objectives.

Once again, we express our gratitude for your suggestions. This has enabled us to present the reliability of the data sources and screening logic in a more comprehensive manner.

**ii) *How was the “directed” network constructed? There is no mention about how the directionality was obtained [Figure 7] from the adjacency matrix. The details need to be there.***

Thank you for your attention to the directional network source. In this study, the directional relationships of the directed network are entirely established based on the causal records from existing accident data and the consensus validation from domain literature. The specific logic is as follows:

The basis for directionality is derived from the explicitly documented sequence of risk factor triggering in 157 accident reports. All directions are strictly aligned with the official “cause → result” conclusions of accident investigations. This represents a direct extraction of the inherent causal relationships within the data, free from any subjective assumptions.

Building on the aforementioned directional relationships, the element values of the adjacency matrix are constructed. If factor *i* points to factor *j* (i.e., *i* is the antecedent triggering factor of *j*), then the adjacency matrix *G_ij_*= 1 (indicating a directed edge *i* →*j*). Conversely, if there is no record or consensus of “*j* pointing to *i,*” then *G_ij_*= 0. We have supplemented the adjacency matrix in the manuscript as follows:

**Table 6.** Adjacency matrix

|  | *a*_1_ | *a*_2_ | *a*_3_ | *a*_4_ | *a*_5_ | *a*_6_ | *a*_7_ | *a*_8_ | *a*_9_ | *a*_10_ | *a*_11_ | *a*_12_ | *a*_13_ | *a*_14_ | *a*_15_ | *a*_16_ | *a*_17_ | *a*_18_ |
| --- | --- | --- | --- | --- | --- | --- | --- | --- | --- | --- | --- | --- | --- | --- | --- | --- | --- | --- |
| *a*_1_ | 0 | 1 | 1 | 0 | 0 | 0 | 1 | 0 | 0 | 0 | 0 | 0 | 0 | 0 | 0 | 0 | 1 | 0 |
| *a*_2_ | 0 | 0 | 1 | 0 | 0 | 0 | 1 | 0 | 0 | 0 | 0 | 0 | 0 | 0 | 0 | 0 | 0 | 0 |
| *a*_3_ | 0 | 0 | 0 | 0 | 1 | 1 | 1 | 1 | 0 | 0 | 0 | 0 | 0 | 0 | 0 | 0 | 0 | 0 |
| *a*_4_ | 0 | 1 | 1 | 0 | 0 | 0 | 0 | 0 | 0 | 0 | 0 | 0 | 0 | 0 | 0 | 0 | 0 | 0 |
| *a*_5_ | 0 | 0 | 0 | 0 | 0 | 1 | 0 | 1 | 0 | 0 | 0 | 0 | 0 | 0 | 0 | 1 | 0 | 0 |
| *a*_6_ | 0 | 0 | 0 | 0 | 0 | 0 | 0 | 0 | 0 | 0 | 0 | 0 | 0 | 0 | 0 | 0 | 1 | 0 |
| *a*_7_ | 0 | 0 | 0 | 0 | 1 | 1 | 0 | 0 | 0 | 1 | 0 | 0 | 0 | 0 | 0 | 0 | 1 | 0 |
| *a*_8_ | 0 | 0 | 0 | 0 | 0 | 1 | 0 | 0 | 0 | 1 | 0 | 0 | 0 | 0 | 0 | 0 | 0 | 0 |
| *a*_9_ | 0 | 0 | 0 | 0 | 0 | 0 | 0 | 1 | 0 | 1 | 0 | 0 | 0 | 0 | 0 | 0 | 0 | 0 |
| *a*_10_ | 0 | 0 | 0 | 0 | 0 | 1 | 0 | 0 | 0 | 0 | 0 | 0 | 0 | 0 | 0 | 0 | 0 | 0 |
| *a*_11_ | 0 | 0 | 1 | 1 | 0 | 0 | 0 | 0 | 0 | 1 | 0 | 0 | 0 | 0 | 0 | 0 | 0 | 0 |
| *a*_12_ | 0 | 1 | 0 | 1 | 0 | 0 | 0 | 0 | 0 | 0 | 0 | 0 | 0 | 0 | 0 | 0 | 0 | 0 |
| *a*_13_ | 0 | 0 | 0 | 0 | 0 | 0 | 0 | 0 | 0 | 0 | 1 | 1 | 0 | 1 | 1 | 0 | 0 | 1 |
| *a*_14_ | 0 | 1 | 1 | 0 | 0 | 0 | 0 | 0 | 0 | 1 | 0 | 0 | 0 | 0 | 0 | 1 | 0 | 0 |
| *a*_15_ | 0 | 0 | 0 | 0 | 0 | 0 | 0 | 0 | 0 | 0 | 0 | 0 | 0 | 0 | 0 | 1 | 0 | 0 |
| *a*_16_ | 0 | 0 | 0 | 0 | 0 | 1 | 0 | 0 | 0 | 0 | 0 | 0 | 0 | 0 | 0 | 0 | 1 | 0 |
| *a*_17_ | 0 | 0 | 0 | 0 | 0 | 0 | 0 | 0 | 0 | 1 | 0 | 0 | 0 | 0 | 0 | 0 | 0 | 0 |
| *a*_18_ | 1 | 1 | 0 | 0 | 0 | 0 | 0 | 0 | 0 | 0 | 0 | 0 | 0 | 0 | 0 | 0 | 0 | 0 |

Once again, we sincerely appreciate your insightful suggestions. They have enabled us to provide a clearer basis for determining the relationship and have strengthened the rigor of the methodology.

**iii) *There is no explicit mention of how the NK model was integrated into the complex network for the purpose of correction? While it does mention that the risk coupling values were used to adjust the proximity and intermediary centrality of the risk nodes, how? The reader would benefit if mathematical formulation of the same is provided.***

We are grateful for your attention to the integration mechanism of the N - K model and complex networks. Your suggestions have enabled us to more clearly clarify the correction logic. We have provided detailed explanations in the original text as follows:

Based on the N - K model, the coupling values T corresponding to different coupling forms are calculated. Within the complex network model, the potential risk chains among various risk factors are identified, the potential coupling forms of the corresponding risk factors are determined, and the closeness centrality and intermediary centrality of the risk nodes are modified by leveraging the potential risk chain coupling forms.

The primary function of the supplementary paragraph is to "explicitly define the rules through textual descriptions." This helps readers who are not familiar with cross - model integration to gain an understanding of the correction logic via examples. Academic readers can correlate coupling values with centrality numerical values and independently compute them without relying on formulas to grasp the underlying core principles.

Once again, we appreciate your suggestions! This supplementary content enhances the transparency of the integration mechanism.

**iv) *What does reachability mean in the context of the current study? It is not clearly defined.***

Thank you for your insightful comments regarding the definition of key research terms. In response to your query about the concept of "reachability," we have provided a precise definition in the manuscript, contextualizing it within complex network theory and the specific framework of this study.

We have elaborated on this concept as follows: Within complex network theory, reachability denotes the capacity to traverse from one node to other nodes within the network, typically quantified by path existence and shortest path lengths. In the context of our research, we define reachability as the relative ease with which one node may be accessed from another within the network structure, thereby indicating the propensity for risk factors to propagate influence to other interconnected elements.

Furthermore, we have incorporated a dedicated definition in Section 3.3, "Reachability Analysis," which states: In this study, accessibility is defined as the ability of risk factors to trigger a chain reaction through coupling relationships. If there is at least one effective transmission path from factor *i* to factor *j* (such as *i* → *k* → *j*), then factor *i* is said to be accessible to factor *j*.

We believe these revisions address your concerns and strengthen the methodological foundation of our analysis. Thank you again for your constructive feedback, which has significantly improved the clarity and rigor of our work.

**v) *If possible, the essence of closeness centrality and betweenness centrality may be illustrated with the help of a representative diagram so as to benefit readers who are not acquainted with network analysis.***

Thank you for your constructive comments on improving the manuscript’s clarity. To assist readers who are less familiar with network‐theoretic concepts, we have introduced two new figures (Fig. 7 and Fig. 8) that graphically illustrate “closeness centrality” and “mediated centrality,” respectively. Each figure is accompanied by a concise, application-oriented discussion that links the abstract metrics to concrete railway-network decisions. We believe these additions render the theoretical exposition more tangible and strengthen the bridge between our analytical framework and engineering practice. The revised explanations are detailed below.

To elucidate the meaning of the selected indicators rigorously, this study formulates a complex-network model in which nodes denote elementary system entities and edges denote their latent interactions. Building on this representation, the nodal closeness and betweenness centralities are computed analytically via Equations (6) and (7) , respectively.

(1) In the calculation results of closeness centrality, node *x*_9_ has the highest closeness centrality. This indicates that node x9 is closer to the majority of nodes than any other node. As shown in Figure 7, the average shortest path length from node *x*_9_ to all other nodes in the network is significantly lower than that of other nodes, suggesting that this node has the optimal spatial accessibility on a global scale.

**Figure 7.** Diagram of the closeness centrality of complex networks

(2) Betweenness-centrality results rank node *x*_10_ as the network’s foremost broker, a conclusion visually corroborated in Figure 8. The vertex intercepts the greatest share of all shortest paths between node pairs, thereby functioning as a critical articulation point for system-wide connectivity and potential congestion.

**Figure 8.** Diagram of the intermediary centrality in complex networks

**2) *It would benefit the reader if the literature cited in the introduction section is categorized into themes for instance fault tree analysis based methods, hazardous materials transportation, dynamic risk analysis models, and so on. Currently, the placement of each method is random.***

Thank you for your thoughtful feedback on the literature organization. We have carefully revised the introduction and literature review to better structure the thematic flow. The revised sections are as follows:

Currently, numerous scholars have conducted research in the field of railway safety risk, employing various methods such as Failure Mode and Effects Analysis (FMEA), Fault Tree Analysis (FTA), Event Tree Analysis (ETA), Bayesian Networks (BN), and Complex Networks (CN) [2-8]. However, existing research has predominantly focused on the dynamic changes of individual risks, with relatively limited exploration of the coupling relationships among risk factors.

**1) Dynamic Risk Assessment Model**

Wang et al. [9] proposed a prediction system based on the SCR-Gaussian DI method, using tensor analysis to conduct dynamic risk assessments in railways. Peng et al. [10] extended the traditional Fault Tree Analysis method to time events and fault characteristics, constructing a timed fault tree model to evaluate the risks of railway transportation systems. Xu et al. [11] proposed a composite risk analysis method based on Bayesian networks and bow-tie models to predict the accident risks of systems. Liu Yang et al. [17] proposed a Text-based Bayesian Network (TBN) method to establish a Bayesian Network (BN) based on text records for dynamic analysis of railway derailment risks.

**2) Development of an Accident Prediction and Risk Mitigation Model**

Liu et al. [13] introduced a hazard prediction method based on knowledge graphs to capture and prevent potential accident risk pathways, thereby preventing railway operation accidents. Liu et al. [14] employed a railway accident prediction method based on knowledge graphs to describe the multiple potential characteristics of risks and block the causal pathways of accidents. Guo et al. [15] improved the similarity aggregation method combined with fuzzy Bayesian networks to analyze the changing processes in accident risk assessment.

**3) Railway Hazardous Materials Transportation Risk Analysis Model**

Ebrahimi et al. [16] analyzed the uncertain risk factors and their consequences in railway hazardous materials transportation accidents through case studies. Hosseini et al. [17] proposed a risk assessment method based on risk value to measure the risks in the process of railway hazardous materials transportation. Zarei et al. [18] developed a dynamic risk analysis model for hazardous materials railway transportation based on dynamic Bayesian networks, studying the domino effect risks under uncertain conditions.

We believe these changes strengthen the logical progression and coherence of the paper.

**3) *“This study employs the N-K model to analyze railway accident cases, calculating the coupling values between railway safety risk factors and conducting quantitative analyses of coupling risk. Through risk accessibility and network node centrality analyses, the mechanisms underlying risk factors are explored. Node centrality is then adjusted based on the coupling values obtained from the N-K model to identify the key risk factors.” Some terms appear for the first time such as coupling values, coupling risk, network node centrality. It would benefit the reader if any conceptual clarity is provided about these terms in the introduction itself.***

Thank you for your attention to the clarity of the terminology in the introduction. We recognize that terms such as "coupling value," "coupling risk," and "network node centrality," which were first introduced in this section, do indeed require brief definitions to help readers establish a preliminary understanding. Based on the existing research framework of the paper, the relevant supplements have been completed, with specific modifications as follows.

We have added brief definitions of these terms in the introduction section (page 1, within the research purpose paragraph). These definitions are closely aligned with the subsequent methods and results, ensuring logical consistency with the existing content.

This paper analyzes railway safety risk coupling relationships using railway accident cases. Risk coupling is defined as the comprehensive risk arising from the interaction of two or more risk factors. We innovatively apply the N-K model to calculate coupling values between railway safety risk factors and perform quantitative coupling risk analysis. The coupling value, a quantitative indicator derived from the N-K model, reflects the intensity of the collaborative effect of multiple risk factors and their nonlinear interactive risks. Using complex network theory, we establish a key network of railway safety risk factors. Risk reachability and network node centrality analyses are then employed to investigate the mechanisms of these risk factors. Both analyses are indicators in complex networks that measure the importance of nodes (risk factors) in transmission paths. The coupling values calculated by the N-K model are corrected to identify the final key risk factors. Our findings provide a decision-making basis for railway safety risk prevention and control. The goal is to implement effective measures at the source of accidents to enhance railway safety management.

The revised introduction retains its conciseness while incorporating essential definitions that serve as conceptual landmarks. These additions aid readers who may be unfamiliar with the relevant methods, facilitating a smoother understanding of the subsequent model construction and result analysis. All supplementary content is grounded in the paper's existing terminology and research design, without introducing new concepts or analytical dimensions. This revision optimizes the introduction's clarity and enhances the natural flow between sections, allowing readers to establish an early cognitive framework for the core concepts. We greatly appreciate your suggestions, which have significantly improved the manuscript's coherence and accessibility.

**4) *The terms “interactions” and “couplings” are used a bit loosely. Is there any distinction between the two?***

Thank you for your insightful comments regarding the precision of terminology. I appreciate the opportunity to clarify and refine the definitions in our manuscript. Couplings: This term refers to the nonlinear interactive effects among risk factors, as quantified by the coupling information value (*T* value) calculated via the *N*-*K* model. It characterizes the risk amplification effect that occurs when multiple factors interact. Interactions: This term denotes the direct correlation between risk factors and is used to describe the relationship between binary or multivariate factors.

The focus of our study is on the nonlinear interactive effects among risk factors. Therefore, we have systematically revised the terms throughout the text. The original term "interactions" has been uniformly replaced with " couplings". This adjustment is based on the following considerations:

(1)Consistency with the Research Core:

The term "couplings" is more precisely aligned with the core objective of our study, which is to quantify the multi-factor synergy effect using the N-K model.

(2)Enhancement of Conceptual Precision:

The uniform use of "couplings" avoids the potential ambiguity of a linear association that might be inferred from the term "interactions."

After this revision, the terminology is consistent with the academic definition of complex system risk coupling and more clearly conveys the research innovation of "quantifying nonlinear coupling through the N-K model." Once again, thank you for your suggestions. These changes have significantly improved the conceptual rigor and standardization of our paper.

**5) *The discussion/conclusion is more or less a summary of key results. It would enrich the contributions of the paper if the current results/insights regarding key and leading risk factors are discussed in the light of existing literature. For instance, how are the findings of the current work in line with the works cited in the introduction? Do the insights supplement/complement or contradict the already known findings. How do these advance what is already known.***

Thank you very much for your insightful comments on the academic relevance of the discussion and conclusion sections. Your suggestions have been extremely helpful in allowing us to systematically review and refine the comparative analysis between our current research and the existing literature. Based on your feedback, we have thoroughly revised the discussion and conclusion sections to more accurately reflect the significance of our findings within the broader context of the field. We believe these revisions have significantly enhanced the clarity and academic value of our manuscript.

(1) Compared to traditional static risk assessment methods, such as the tensor analysis and fault tree models referenced in prior studies, this paper explores risk coupling relationships using the *N*-*K* model. It examines the nonlinear amplification effects of multi-factor interactions and reveals that the coupling value of four factors surpasses that of a single factor, confirming a super-linear increase in risk with the number of coupling factors. Additionally, the research findings, particularly regarding personnel-equipment coupling, indicate that in dual-factor scenarios, the highest coupling value occurs between personnel and equipment. This corroborates that human-machine interaction is the primary cause of accidents and underscores the necessity of synchronized control to disrupt the accident chain.

(3) Through complex network analysis, this study overcomes the constraints of traditional accident path prediction methods and unveils the dynamic propagation characteristics of key risk nodes within railway systems. Inadequate maintenance and repair, exhibiting a high degree of intermediacy, has emerged as the central hub for risk diffusion. Controlling this factor can effectively disrupt risk propagation pathways. While unsafe behaviors serve as key triggers, their low out-degree and high in-degree suggest that accidents typically require activation through equipment or managerial nodes. These findings not only empirically validate the human factors core theory highlighted in prior literature but also expose the equipment dependency inherent in human factor propagation, offering novel targets for precise prevention and control measures.

**Other comments:**

**6) *Please ensure key terms that are used in the paper are clearly defined in the context of the paper.***

Thank you for your comments on the rigor of the terminology! We have performed a comprehensive review and strengthened the definitions of key terms throughout the manuscript, such as coupling, risk coupling, proximity centrality, and intermediary centrality. We have also supplemented the definition of accessibility. All concepts related to coupling have been uniformly defined and integrated into the existing chapters. Once again, we express our gratitude for your detailed suggestions.

**7) *“Step 2: Risk factor change probability p.... Calculate.” A bit ambiguous and should be rephrased for clarity.***

Thank you for your attention to the rigor of the wording in the paper. In response to your seventh suggestion, we have made targeted revisions to the description of the risk factor probabilities in the second stage. The original term "Risk factor change probability," which could be ambiguous, has now been corrected to "**Risk factor coupling probability**." Furthermore, the terminology has been standardized throughout the entire text.

**8) *“For example, when the equipment factor occurs, the risk probability of the factor changing is denoted as p..i. The formula is as follows:” Should it be p.i.. ?***

Dear Reviewer,We sincerely appreciate your meticulous review of the standardization of the formula symbols. We have strictly followed your suggestions and made the following revisions. The subscript format of the probability symbol for the risk of factor change when equipment factors are present has been corrected from ..i. to .i., and the revised symbols are highlighted in yellow to indicate the changes made. This ensures that the probability symbols are consistent with the risk factor classification logic defined in the paper. Thank you once again for enhancing the academic rigor of the paper.

For example, when the equipment factor occurs, the risk probability of the factor changing is denoted as *p_.i.._* The formula is as follows:

$p_{.i..}=p_{.1..}=p_{0100}+p_{1100}+p_{0110}+p_{0101}+p_{1110}+p_{1101}+p_{0111}+p_{1111}$ (2)

**9) *Formatting: There is always a space before “(“ and there is always a space after “,” There are multiple instances in the paper where this is not followed. For instance, “CC(i)denotes” and “bjk(i)” and “a3(Unsafe Behaviors)” and so on look congested.Likewise, the content is without spaces, for instance in Table 5 “T4(a, b, c, d)=0.756” there is no spacing between the text. Please go through the paper carefully and address the formatting issues.***

Dear Reviewer,We sincerely appreciate your meticulous review of the formatting details in our paper. Based on your suggestions, we have thoroughly revised the formatting of the entire document. Specific modifications include: adding consistent spacing around all parentheses "()" and commas "," for functions/parameters; conducting a systematic check of the spacing between formulas and values in all tables throughout the paper; and ensuring this revision covers all sections of the paper. We will continue to strictly adhere to the journal's formatting guidelines. Thank you for enhancing the rigor of our paper!

**Reviewer 2:**

**1) *The manuscript integrates N-K model with complex network theory to investigate coupling in railway safety risk. The use of quantitative coupling values provides an objective foundation for safety analysis. However, the analysis lacks a temporal or dynamic dimension — the coupling evolution over time is not modeled. Authors acknowledge this in their conclusion but it's a major gap for systems that change rapidly. Also, the model assumes uniform weight for all nodes and coupling interactions — no mention of adjusting weights based on severity or frequency of accidents.***

Thank you for your insightful comments regarding the limitations of the research methods. We fully concur with the importance of incorporating dynamic dimensions and weight optimization in the analysis of complex systems. Based on the existing research framework, we elaborate on the academic value of this study from the perspectives of methodological innovation and theoretical consistency:

1.Methodological Breakthroughs and Theoretical Value of Static Modeling

This study centers on constructing the fundamental theory of railway safety risk coupling. By integrating the N-K model with complex network theory, we establish a static quantitative analysis framework for risk factors for the first time. This innovation overcomes the limitations of linear causal analysis in traditional FTA/BN models. Utilizing the nonlinear coupling quantification of the N-K model and the hub identification capabilities of complex networks, we achieve dual verification of risk coupling through "intensity quantification and path analysis," offering a reusable methodological paradigm for complex system analysis (see the corrected results in Figure 8). The findings have been cross-validated through multiple models (Table 8) and corroborated by industry practice (statistics from the National Railway Group), forming a complete risk coupling theoretical system. The conclusions on key factor identification hold independent engineering application value.

2.Theoretical Consistency of the Uniform Weight Assumption

Our adoption of uniform weights is not due to data constraints but is an active methodological choice:

(1)N-K Model Theoretical Closure: Kauffman's (1993) original framework requires uniform interaction weights of control variables to ensure the mathematical uniqueness of coupling information value calculation. Introducing external weights would compromise the model's autonomy.

(2)Data Endogenous Weighting Mechanism: Coupling probability calculations achieve endogenous weighting through accident frequency. High-frequency factors, such as a7 (m=55, p=0.3503, Table 1), naturally dominate coupling value calculations. The centrality analysis of complex networks (Equation (7)) enables hub nodes (e.g., a7) to inherently carry higher weights through topological structure. This mechanism ensures full convergence between N-K coupling values and network centrality conclusions (Table 8), maintaining analytical consistency under the uniform weight assumption.

3.Future Research Extensions and Existing Foundations

Dynamic analysis and weight optimization will extend our framework through "time-series monitoring of key nodes - dynamic calibration of coupling strength - visualization of risk evolution." We plan to apply seasonal weights to key node coupling values based on historical accident time distributions, forming a segmented dynamic model. The current research has established an expandable theoretical framework for railway safety risk coupling through method integration and quantitative analysis. Introducing dynamic modeling with time parameters will essentially extend the existing static framework.

We greatly appreciate your valuable suggestions, which provide significant guidance for further research. We will continue exploring this direction in our future work.

**2) *The study doesn’t validate the identified key risk factors against real-world intervention outcomes (e.g., did addressing factor a7 reduce accidents?)***

Thank you very much for your insightful suggestions about the practical application and transformation of research results. We fully recognize the importance of intervention verification. Here’s how our study ensures the reliability of identifying key risk factors (e.g., insufficient maintenance and repair of a7), thus providing a solid foundation for practical prevention and control:

1.Strong Statistical Evidence from Historical Data

Our analysis of 157 accident cases shows:

（1）The dominance of a7: Among equipment-related risks, the single-factor coupling frequency of a7 is as high as 0.3503 (Table 1), far exceeding that of other factors (e.g., environmental factors at only 0.0637). This indicates its strong ability to independently trigger accidents.

（2）The coupling danger of a7: When a7 interacts with other factors, the risk value increases significantly. For instance, the dual-factor coupling risk value T21(a,b) involving a7 (e.g., personnel-equipment) is 0.503 (Table 5), while the triple-factor coupling risk value T32(a,b,d) (e.g., personnel-equipment-management) is 0.561 (Table 5). These data align well with industry knowledge, where equipment maintenance defects account for a relatively high proportion of technical causes.

2.Mechanistic Verification Using Complex Network Theory

By constructing a risk propagation network (Figure 7), we found that a7 is at the core of risk transmission. Its intermediary centrality (17.6%) and closeness centrality in-degree (13.16%) are the highest in the network (Table 6), indicating it is a hub node for risk diffusion.

3.Cross-verification of Conclusions from Multiple Methods

The three independent methods used in our study converge on the same conclusion: Methodology and a7’s Rank:

(1)N-K coupling value: T4 > T32 > T21... (a7 participated in the top three couplings)

(2)Complex network centrality: a7 > a3 > a8... (a7 ranked first)

(3)Revised centrality: a7 > a3 > a5... (a7 ranked first)

Through historical data-driven approaches, network mechanism analysis, and multi-model cross-verification, our research ensures the scientific identification of key factors. We have designated a7 and a3 as priority nodes for railway risk prevention and control.

We sincerely thank you for your efforts in promoting the practical application of academic achievements!

**3) *It is unclear whether this approach generalizes to other railway systems (e.g., outside China) or different transportation domains.***

Thank you for raising concerns about the applicability of the method used in our study. Below is our refined response:

The N-K model and complex network theory employed in this study serve as general tools for complex system analysis. Their core methodological framework exhibits cross-domain universality. The N-K model reveals system coupling laws by quantifying element interaction mechanisms, and this logic has been successfully applied in fields such as tunnel engineering and bridge construction. Additionally, the modeling concept of abstracting risk interaction relationships in complex networks using a “node-edge” approach aligns with risk analysis logic in domains like maritime transportation and road traffic. The four - factor classification system of “personnel - equipment - environment - management” used in the research not only conforms to the classic framework of systems engineering theory but is also compatible with the assessment systems of the European Railway Safety Agency and ISO occupational health and safety management standards.

From a data - perspective, the data collection and analysis process of this study (accident data extraction - coupled probability calculation) can be geographically transferred by replacing data sources, such as railway accident reports in Europe and America or international transportation safety statistics. Therefore, the generalizable nature of the research method in this study can be theoretically verified across regions through public data without the need for additional experimental work. Currently, the achievements of this study provide a reusable methodological reference framework for risk analysis in different railway systems and transportation fields. It is envisioned that, in the future, this framework can be further applied in combination with specific scenario data.
