# Supplementary material for: Investigating the coupling relationships of railway safety risks using the N-K model and complex network theory
Source: PLoS One. 2025 Sep 22;20(9):e0332948. doi: 10.1371/journal.pone.0332948 (PMC12453224; doi:10.1371/journal.pone.0332948)
Supplement: Attachment — Submitted filename: Response_to_Reviewers_auresp_2.docx [file pone.0332948.s003.docx]

Dear editor and reviewers,

We appreciate the opportunity to revise our manuscript titled " Investigating the Coupling Relationships of Railway Safety Risks Using the *N*-*K* Model and Complex Network Theory " and are grateful for the insightful comments provided by the reviewers. Those comments are all valuable and very helpfu for revising and improving our paper, as well as the important guiding significance to our researches. In the following, we have provide detailed responses to each of the reviewers' comments. The reviewers' comments are presented in *italic font*, with specific issues numbered. Our responses are presented in normal font, while the modifications and additions to the manuscript are marked in **blue font**. Additionally, in the revised manuscript, the modified charts, formulas, and symbols are highlighted in **yellow**.

**Reviewer 1:**

**1) *While the clarification of the following comment “How was the “directed” network constructed? There is no mention about how the directionality was obtained [Figure 7] from the adjacency matrix. The details need to be there.” has been provided in the comments document, it has not been included in the manuscript (even though adjacency matrix has now been included). It would be helpful to include a few sentences in the manuscript as well.***

Thank you for pointing out the issue that "the details of directed network construction were not included in the manuscript". After verification, previously we only explained in the opinion response document how to obtain the directionality of Figure 7 from the adjacency matrix, but we did not integrate this key information into the main text of the manuscript. There is indeed an incomplete presentation of information. We sincerely apologize for this. In the revised manuscript section, we have added the following content:

By collecting a large number of railway accident cases and analyzing their causes, we have summarized the adjacency matrix of risk factors affecting railway safety ,The value rule for the elements in the adjacency matrix (Table 6) is as follows: If factor *i* points to factor *j* (i.e., *i* is the antecedent triggering factor of *j*), then the adjacency matrix *G_ij_* = 1 (indicating a directed edge *i* →*j*). Conversely, if there is no record or consensus of “*i* pointing to *j*,” then *G_ij_*= 0. Based on this matrix, a complex network diagram of railway safety risk factors has been constructed, as shown in Figure 8.

**2) *“The centrality values of the risk factors are presented in Table 6”. Table 6 is an adjacency matrix. Do you mean Table 7?***

Thank you for highlighting the citation error. We have corrected the sentence to read: “The centrality values of the risk factors are presented in Table 7.” In addition, we have systematically reviewed every table citation in the manuscript to ensure complete consistency between textual references and the corresponding tabulated data, thereby eliminating any further discrepancies.

**3) *Figures 7 and 8 can be replaced by a single figure because they are the same except for the color of two nodes. A single figure with different colors for the two referred nodes may be used.***

Thank you for your valuable suggestion regarding Figures 7 and 8. We agree that the two panels conveyed redundant information; the only distinction was the node colouring. Accordingly, we have consolidated the two figures into a single, revised Figure 7. The updated figure overlays both centrality metrics: node *x*_9_ is highlighted in red (closeness centrality) and node *x*_10_ in blue (betweenness centrality). This refinement eliminates visual redundancy, reduces the total number of figures, and allows readers to apprehend the centrality contrasts between the two nodes at a glance.

Among the computed centrality metrics, node *x*_9_ attains the highest closeness centrality, signifying its minimal average geodesic distance to all other nodes in the network. Concurrently, node *x*_10_ records the greatest betweenness centrality, functioning as the single most critical articulation point for inter-node traffic. Figure 7 visualizes these findings: red-marked *x*_9_ exhibits a markedly shorter mean shortest-path length, indicating superior global accessibility; conversely, blue-marked *x*_10_ participates in the largest fraction of shortest paths, establishing itself as the primary structural pivot. However, this pivotal role also renders *x*_10_ a potential congestion bottleneck under high-demand scenarios.

**Figure 7.** Diagram illustrating the concept of closeness centrality and betweenness centrality in complex networks

**4) *Table 8 has something written in a language that is not English. Please replace it with English text.***

Thank you for identifying the non-English element in Table 8. We have rectified the oversight: the column heading previously rendered as “潜在耦合形式” now reads “Potential Coupling Forms,” and we have re-examined the entire table to ensure conformity with the manuscript’s English-language standards.

**5) *In the “Construction of the N-K Risk Coupling Model for Railway Systems” section, the headings of step 1 and step 2 convey the same meaning. Please modify Step 2 heading appropriately. Currently, Step 1 reads “Calculation of the Coupling Probability p for Risk Factors.” while Step 2 reads “Risk factor coupling probability p.... Calculate”.***

Thank you for highlighting the redundancy in the headings for Steps 1 and 2 within the “Railway System N-K Risk-Coupling Model Construction” chapter. Upon verification, the original wording—“**Calculation of the Coupling Probability *p* for Risk Factors**” (*Step 1*) and “**Risk factor coupling probability *p_...._* calculate**” (*Step 2*)—was indeed repetitive and risked obscuring the sequential logic.

We have revised Step 2 to read “**Step 2: Coupling probability *p*.... associated with specific risk factors.**” This change clarifies that Step 1 determines the overall probabilities for single-, double-, and multi-factor couplings across the entire risk-factor set, whereas *Step 2* derives the conditional probability contingent on the occurrence of a specific risk factor (e.g., the equipment-factor state-change probability *p_.i.._* referenced in the text). The revised heading thus delineates the analytical progression without modifying the underlying formulas or methodology.

We have also reviewed the remaining step headings in the chapter to ensure consistency and terminological precision.
